# Supplementary material for: Quality Attributes of Cryoconcentrated Calafate (Berberis microphylla) Juice during Refrigerated Storage
Source: Foods. 2020 Sep 18;9(9):1314. doi: 10.3390/foods9091314 (PMC7555764; doi:10.3390/foods9091314)
Supplement: Supplementary file 1 [file foods-09-01314-s001.pdf]

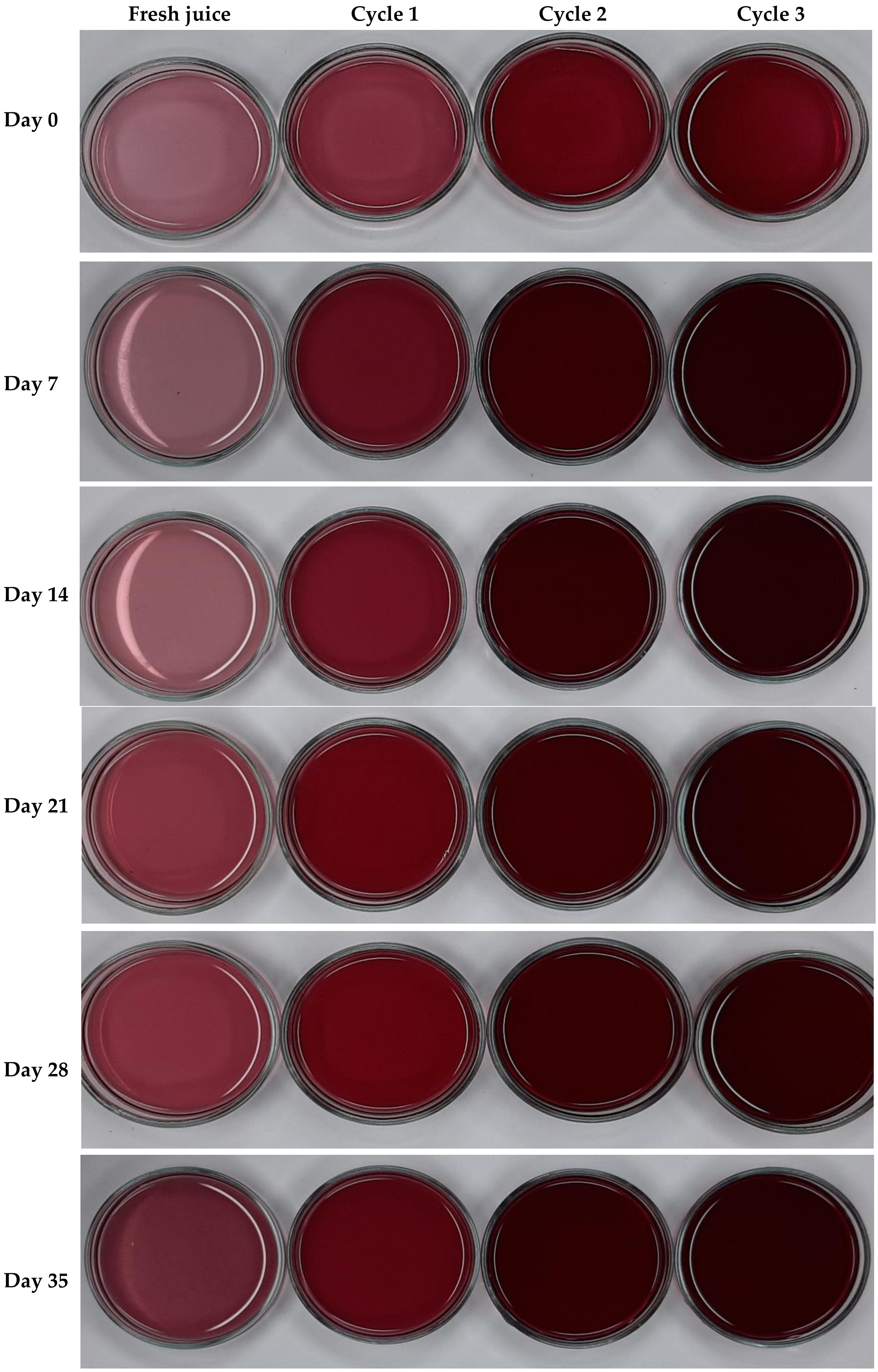

Fig. S1. Visual appearance of fresh juice and concentrate samples at each cycle during storage.

Table S1. Pearson's correlation coefficients (r) between biological active compounds content of fresh calafate juice.

	TPC	TAC	TFC	DPPH	ABTS	FRAP	ORAC
TPC	1.00						
TAC	1.00*	1.00					
TFC	0.99*	1.00*	1.00				
DPPH	0.99*	1.00*	1.00*	1.00			
ABTS	0.97*	0.98*	0.98*	0.99*	1.00		
FRAP	0.97*	0.98*	0.98*	0.99*	1.00*	1.00	
ORAC	0.96*	0.97*	0.97*	0.98*	1.00*	1.00*	1.00

*Significant at 5%.

Table S2. Pearson's correlation coefficients (r) between biological active compounds content (TBC and TAA) obtained by CBCC process (first cycle).

	TPC	TAC	TFC	DPPH	ABTS	FRAP	ORAC
TPC	1.00						
TAC	0.99*	1.00					
TFC	0.98*	1.00*	1.00				
DPPH	0.98*	1.00*	0.99*	1.00			
ABTS	0.98*	1.00*	0.99*	1.00*	1.00		
FRAP	0.97*	0.98*	0.98*	0.98*	0.99*	1.00	
ORAC	0.97*	0.98*	0.97*	0.98*	0.99*	1.00*	1.00

*Significant at 5%.

Table S3. Pearson's correlation coefficients (r) between biological active compounds content (TBC and TAA) obtained by CBCC process (second cycle).

	TPC	TAC	TFC	DPPH	ABTS	FRAP	ORAC
TPC	1.00						
TAC	0.96*	1.00					
TFC	0.91*	0.99*	1.00				
DPPH	0.96*	1.00*	0.99*	1.00			
ABTS	0.94*	1.00*	0.99*	1.00*	1.00		
FRAP	0.94*	0.99*	0.98*	0.99*	0.99*	1.00	
ORAC	0.94*	0.98*	0.97*	0.99*	0.99*	1.00*	1.00

*Significant at 5%.
